# Multifunctional Konjac Glucomannan Film Tuning by Gallic Acid Functionalization

**DOI:** 10.3390/polym18070832

**Published:** 2026-03-28

**Authors:** Lidia Zasada, Marcin Wekwejt, Marta Michalska-Sionkowska, Anna Michno, Anna Ronowska, Karina Fałkowska, Karolina Dulok, Beata Kaczmarek-Szczepańska

**Affiliations:** 1 Laboratory for Functional Polymeric Materials, Faculty of Chemistry, Nicolaus Copernicus University in Toruń, Gagarina 7, 87-100 Torun, Poland; 503555@doktorant.umk.pl (L.Z.); 312510@stud.umk.pl (K.F.); 312508@stud.umk.pl (K.D.); 2 Biomaterials Technology Department, Faculty of Mechanical Engineering and Ship Technology, Gdańsk University of Technology, Narutowicza 11/12, 80-233 Gdansk, Poland; marcin.wekwejt@pg.edu.pl; 3 Department of Environmental Microbiology and Biotechnology, Faculty of Biological and Veterinary Sciences, Nicolaus Copernicus University in Toruń, Lwowska 1, 87-100 Torun, Poland; mms@umk.pl; 4 Department of Clinical Biochemistry, Department of Laboratory Medicine, Medical University of Gdańsk, Skłodowskiej-Curie 3a, 80-210 Gdansk, Poland; anna.michno@gumed.edu.pl (A.M.); anna.ronowska@gumed.edu.pl (A.R.); 5 Institute of Advanced Studies, Nicolaus Copernicus University in Toruń, Wileńska 4, 87-100 Torun, Poland

**Keywords:** konjac glucomannan, gallic acid, functionalization, wound dressing, packing materials

## Abstract

Konjac glucomannan (KG) is a biocompatible polysaccharide with limited functional performance in its native form, motivating modification strategies to enhance its properties. This study investigates the effect of gallic acid (GA) functionalization on the structural, physicochemical, mechanical, antioxidant, and biological properties of KG-based films. FTIR analysis confirmed that GA interacts with KG primarily through non-covalent hydrogen bonding without disrupting the polymer backbone. Modification with GA enabled concentration-dependent tuning of surface energy, roughness, hydration behavior, and water vapor permeability. Mechanical testing revealed a significant increase in stiffness and tensile strength accompanied by reduced elongation at higher GA contents. Antioxidant activity was markedly enhanced even at low GA concentrations. All films exhibited excellent hemocompatibility, while cytocompatibility toward human fibroblasts depended on GA content. Optical analysis indicated moderate color changes without severe discoloration. Overall, GA functionalization effectively improves the functional performance of KG films while preserving polymer integrity. Hence, GA-modified KG films as promising candidates for biomedical applications (like wound dressing) requiring antioxidant activity, controlled hydration, and biocompatibility.

## 1. Introduction

Konjac glucomannan (KG) is a natural polysaccharide widely used in biomedical and food-related applications due to its biodegradability, biocompatibility, and excellent film-forming ability [1]. However, its high hydrophilicity and limited mechanical stability restrict its broader use in advanced functional materials [2]. To overcome these limitations, various chemical modifications (like functionalization) and blending strategies have been explored to improve its physicochemical and biological properties [3].

Phenolic acids are a diverse group of naturally occurring plant secondary metabolites characterized by one or more hydroxyl groups attached to an aromatic ring. They are widely distributed in fruits, vegetables, cereals, and medicinal plants, and are recognized for their potent antioxidant, antimicrobial, and anti-inflammatory properties [4,5,6]. In materials science and biomaterials engineering, phenolic acids have attracted growing attention as natural modifiers and crosslinking agents for various polysaccharides and proteins. Their multiple hydroxyl groups can participate in hydrogen bonding, radical scavenging, and covalent interactions, enabling the development of bioactive materials with enhanced stability and tailored functionalities [7,8,9]. The incorporation of phenolic acids (as well as functionalization) into polymer matrices has been reported to enhance mechanical properties, reduce hydrophilicity, and impart bioactivity, making them highly promising for various medical applications, including wound dressings, drug delivery systems and tissue engineering scaffolds [10,11].

Gallic acid (GA), a naturally occurring polyphenol, is well known for its strong antioxidant, antimicrobial, and crosslinking properties [12]. Hence, GA functionalization of polysaccharide-based films offers a promising strategy to enhance not only their stability and surface properties but also their biological performance, especially in the context of biomedical devices and wound dressings [13]. Moreover, the GA can modulate water uptake, strengthen intermolecular interactions, and introduce additional bioactivity that supports cell adhesion and proliferation, and/or microbial resistance [14].

In this study, GA-functionalized KG-based films were prepared and comprehensively characterized to evaluate their suitability for biomedical and packaging applications. A wide range of physicochemical and biological assessments were employed, including Fourier-transform infrared spectroscopy, contact angle and surface free energy analysis, atomic force microscopy, and antioxidant testing using the DPPH radical scavenging assay. Moreover, water content, mechanical strength, water vapor permeability rate, and colorimetric parameters were determined. Finally, in vitro biocompatibility was investigated using human red blood cells and human fibroblasts.

## 2. Materials and Methods

### 2.1. Chemicals

For film preparation: konjac glucomannan (KG, Pol-Aura, PL), gallic acid (GA, Pol-Aura, PL), and acetic acid (AA, Pol-Aura, PL) were used in experimental studies.

For physicochemical studies: diiodomethane (99%) was supplied from Sigma Aldrich (Poznań, Poland), and glycerin was purchased from Avantor Performance Materials Poland S.A. (Gliwice, Poland).

For biological studies: Triton™ X-100, acid citrate dextrose solution, MTT reagent (3-(4,5-dimethylthiazol-2-yl)-2,5-diphenyltetrazolium bromide), sodium dodecyl sulfate (SDS), N,N-dimethylformamide (DMF), Ham’s F12 Medium (phenol red-free), Dulbecco’s Modified Eagle Medium (DMEM), L-glutamine, streptomycin, penicillin, fetal bovine serum (FBS), and geneticin (G418 sulfate) were purchased from Merck (Poznan, Poland), unless otherwise stated.

### 2.2. Material Preparation

**Figure 1:**
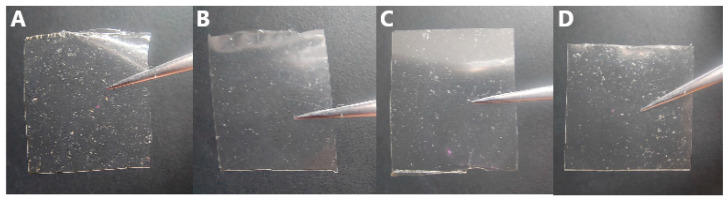
The images of the obtained films. (**A**) 100KG; (**B**) 99.75KG/0.25HA; (**C**) 99.50KG/0.50GA; (**D**) 99.00KG/1.00GA.

KG and GA were separately dissolved in 0.1 M acetic acid at 1% concentration. Solutions were mixed in the ratios 99.75/0.25, 99.50/0.50, and 99.00/1.00 (*w*/*w*%; based on preliminary studies) for 2 h at room temperature on a magnetic stirrer to ensure homogeneity. After stirring, the solutions were poured into plastic holders with a volume of 40 mL and a surface area of 10 cm × 10 cm, and thin films were obtained by solvent evaporation in room conditions for 48 h. KG-based films without GA were used as control samples for comparison (Table 1; Figure 1).

### 2.3. Attenuated Total Reflect–Fourier Transform Infrared (ATR-FTIR)

The infrared spectra of the films were obtained at room temperature under ambient conditions using a Nicolet iS5 spectrometer (Thermo Fisher Scientific, Waltham, MA, USA) equipped with an ID7 ATR accessory, (Thermo Fisher Scientific, Waltham, MA, USA). The measurements were performed with a spectral resolution of 4 cm^−1^, with 32 scans over the wavenumber range 4000–500 cm^−1^.

### 2.4. Contact Angle Measurement

The contact angle (n = 5) was determined by observing the profile of a liquid droplet on the surface. The surface free energy, including its polar and dispersive components, was calculated using the Owens–Wendt method based on contact angle measurements with two probe liquids: water and diiodomethane. The measurements were conducted at a constant temperature using a goniometer equipped with a drop shape analysis system (DSA 10 Control Unit, Krüss, Hamburg, Germany).

### 2.5. Atomic Force Microscopy (AFM)

Surface roughness analysis (n = 3) was performed at room temperature in ambient air using a NanoScope IIIa MultiMode Scanning Probe Microscope (Veeco Metrology, Inc., Santa Barbara, CA, USA), operating in tapping mode. The root-mean-square roughness (Rq) and arithmetic mean roughness (Ra) were calculated using Nanoscope Analysis v6.11 software.

### 2.6. DPPH Scavenging Assay

The antioxidant capacity of the films was assessed through the DPPH radical scavenging assay (2,2-diphenyl-1-picrylhydrazyl, 95%) [15]. Film specimens (n = 5) were cut into 1 × 1 cm squares and placed in a 12-well plate. Each well was filled with 2 mL of a 250 µM DPPH methanolic solution. The plates were kept in the dark for 1 h, and all experiments were carried out in triplicate. A DPPH solution without films was used as the control. After incubation, absorbance was recorded at 517 nm with a UV-1800 spectrophotometer (Shimadzu, Muttenz, Switzerland). Radical scavenging activity (RSA%) was then determined using the following equation:

(1) $$RSA\%=\frac{{Abs}_{DPPH}-{Abs}_{PB}}{{Abs}_{DPPH}}*100$$

where

Abs_DPPH_ is the absorbance of the DPPH solution without contact with the material being tested;

Abs_PB_ is the absorbance of the DPPH solution after contact with the material being tested.

### 2.7. Water Content

The water content of the films was determined by gravimetric oven-drying. Initially, the specimens (n = 5) were weighed and subsequently placed in an oven set at 105 °C. The drying process continued until a constant mass was achieved, ensuring complete moisture removal. The water content was then calculated and expressed as the amount of water (in grams) per 100 g of dry sample.

### 2.8. Mechanical Properties

The mechanical properties of the films were analyzed using a Shimadzu EZ-Test EZ-SX machine (Kyoto, Japan). Specimens (n = 10) were clamped and stretched at a rate of 5 mm/min. Young’s modulus, maximum tensile strength and elongation at break were derived from the stress–strain curve within the linear region (0.3–1.5 N) using Trapezium X Texture software (version 2.0, Kyoto, Japan).

### 2.9. Water Vapor Permeation Rate (WVPR)

The water vapor permeability rate (WVPR) was measured using a gravimetric method. Containers with a 5 cm diameter were filled with anhydrous calcium chloride (m_0_) as a desiccant and sealed at the top with the test films (n = 5). The assemblies were maintained under ambient conditions for 24 h, after which the weight gain of the desiccant, corresponding to absorbed water vapor, was recorded. WVPR was then calculated and expressed as g/cm^2^/h.

### 2.10. Differences in Color and Whiteness Index

The color variations in the obtained films (n = 5) were studied using the colorimeter (Corneometer CL 400, Courage + Khazaka electronic GmbH; Cologne, Germany). The parameters, such as L (lightness), a (representing the color range from green to red), and b (representing the color range from blue to yellow), were evaluated [16]. The total color difference (ΔE) and whiteness index (WI) were then calculated using Equations (2) and (3), as follows:

∆E = (∆L^2^ + ∆a^2^ + ∆b^2^)^0.5^ (2)

WI = 100 − ((100 − L)^2^ + a^2^ + b^2^)^0.5^ (3)

where:

∆L = L − L_0_; ∆a = a − a_0_; ∆b = b − b_0_;

L—the component describing lightness;

a—the color ranging from green (−a) to red (+a);

b—the color ranging from blue (−b) to yellow (+b);

L_0_; a_0_; b_0_—color values for the white background, L_0_ (lightness), a_0_ (redness/greenness), and b_0_ (yellowness/blueness).

### 2.11. In Vitro Biocompatibility

The compatibility of the tested films was assessed using human red blood cells (RBCs) and human fibroblasts (BJ; skin fibroblasts; PCS-201-012, ATCC-2522; USA). Prior to testing, all specimens were sterilized by UV irradiation (30 W/m; 2 × 30 min).

#### 2.11.1. Hemocompatibility

RBCs were isolated from buffy coats—a byproduct of whole blood fractionation—provided by the Regional Blood Centre in Gdansk (institutional permission no. M-073/17/JJ/11). The whole blood was collected from healthy volunteers in accordance with the Declaration of Helsinki [17] and local ethical guidelines, and the blood components were stored in a standard acid citrate dextrose solution. For hemocompatibility testing, 1.5 mL of a suspension containing 3 × 10^9^ RBCs/mL was incubated with film specimens (0.5 mm × 0.5 mm; 0.013 ± 0.003 mm thickness; n = 4) at 37 °C for 24 h. After incubation, the samples were centrifuged (100× *g*, 3 min, room temperature) to sediment the erythrocytes, and the degree of hemolysis was quantified in the supernatant by measuring absorbance at 540 nm using an Ultrospec 3000 pro spectrophotometer (Amersham-Pharmacia-Biotech, Cambridge, UK). RBCs treated with 0.2% Triton were used as a positive control (100% hemolysis), whereas RBCs incubated without material served as a negative control. Hemolysis values below 2% were considered non-hemolytic according to standard criteria [18].

#### 2.11.2. Cytocompatibility

BJ cells were cultured in a 1:1 mixture of Ham’s F12 and DMEM, supplemented with 1 mM L-glutamine, 0.05 mg/mL of streptomycin, 50 U/mL of penicillin and 10% FBS. Cells were seeded at a density of 12 × 10^3^ cells/well in a 96-well plate and cultured for 24 h at 37 °C in a humidified atmosphere of 5% CO_2_. The cytocompatibility assessment was performed in accordance with the ISO 10993-5 standard [19]. For extract preparation, each specimen (n = 4; surface-to-volume area: 6 cm^2^/mL) was incubated in 2 mL of culture medium for 24 h. The resulting extracts were added to the fibroblast cultures, replacing the original medium. After 48 h of incubation, cell viability was determined using an MTT assay (0.60 mmol/L, 4 h incubation). Formazan crystals formed by metabolically active cells were solubilized in a solution containing 10% SDS and 50% DMF. The absorbance was measured using a microplate reader (Victor, PerkinElmer, Waltham, MA, USA) at 595 nm with a reference at 690 nm. The results were expressed as a percentage of the viability of untreated cells grown on standard tissue culture plastic (TCP), defined as 100%.

### 2.12. Statistical Analysis

The data collected during the study were processed using SigmaPlot 14.0 software (Systat Software, San Jose, CA, USA). The Shapiro–Wilk test was used to assess the normality of the data distribution. Results are expressed as mean values ± standard deviation (SD). Group differences were evaluated using one-way analysis of variance (ANOVA), followed by Bonferroni’s *t*-test for multiple comparisons against the control group. Statistical significance was established at *p* < 0.05.

## 3. Results

### 3.1. Attenuated Total Reflect–Fourier Transform Infrared (ATR-FTIR)

The FTIR spectra of pristine KG and GA-functionalized films revealed no significant changes in the overall profile; however, the subtle shifts listed in Table 2, indicate molecular interactions between KG and GA (Figure S1). All films displayed a broad –OH stretching band at ~3340–3344 cm^−1^, typical of polysaccharides. The slight shift toward higher wavenumbers and the increased band broadening upon functionalization suggest enhanced hydrogen bonding, consistent with GA’s polyhydroxyl structure. The weak band near ~2882–2884 cm^−1^, assigned to aliphatic C–H stretching, remained unchanged, suggesting that the aliphatic backbone of KG was unaffected by GA. In the COO^−^ stretching region, the main band shifted from ~1647 cm^−1^ in pristine KG to ~1639–1640 cm^−1^ in GA-containing films. This variation reflects the influence of carboxyl groups from GA overlapping with KG vibrations. Additional characteristic peaks at ~1373–1374, 1244–1245, 1148–1149, and 1018–1019 cm^−1^, corresponding to glycosidic (C–O–C, C–O) and C–O stretching vibrations, were also observed to exhibit minor shifts or intensity variations after GA functionalization. Taking together, these subtle but consistent changes confirm that GA interacts with KG primarily through hydrogen bonding and van der Waals forces, without generating new covalent bonds. This conclusion aligns with previous studies on polyphenol–polysaccharide complexes, where hydrogen bonding is the predominant mechanism of stabilization [20].

### 3.2. Contact Angle Measurement

The GA functionalization of KG-based films had a noticeable impact on their surface wettability and interfacial properties. The 100KG film exhibited relatively high contact angles for both glycerol (Θ^G^ = 52.98°) and diiodomethane (Θ^D^ = 53.02°), indicating a moderately hydrophilic character with balanced polar and dispersive interactions (Table 3). Upon GA modification, significant changes in contact angles and calculated interfacial tension parameters were observed, depending on GA concentration.

At low GA concentration (0.25–0.50 wt%), Θ^D^ decreased substantially, while Θ^G^ decreased more moderately. This reduction in Θ^D^ reflects an enhancement in polar interactions at the film surface, most likely due to the introduction of hydroxyl and carboxyl groups from GA. These functional groups are known to increase the density of hydrogen bonding sites, thereby increasing the polar contribution to surface energy. Consequently, the polar component of interfacial tension decreased compared with 100KG (16.35–17.85 mJ/m^2^ vs. 18.01 mJ/m^2^), while the dispersive component increased markedly, reaching the highest value for 0.25 wt% GA (28.39 mJ/m^2^). This suggests that at low concentrations, GA was well-dispersed within the KG matrix, enriching the film surface with functional moieties that promoted a favorable polar–dispersive balance. Interestingly, at 1 wt% GA, both Θ^G^ and Θ^D^ values increased again (51.40° and 50.12°, respectively), approaching those of the 100KG film. This shift was accompanied by a decrease in the dispersive component and an increase in the polar component. Such a trend may be explained by molecular aggregation of GA at higher loadings, leading to partial screening of polar groups at the surface and reduced accessibility of active sites. Aggregation phenomena have been widely reported for polyphenols in polymer matrices, which often results in less efficient surface modification at higher additive levels [21].

### 3.3. Atomic Force Microscopy (AFM)

**Figure 2:**
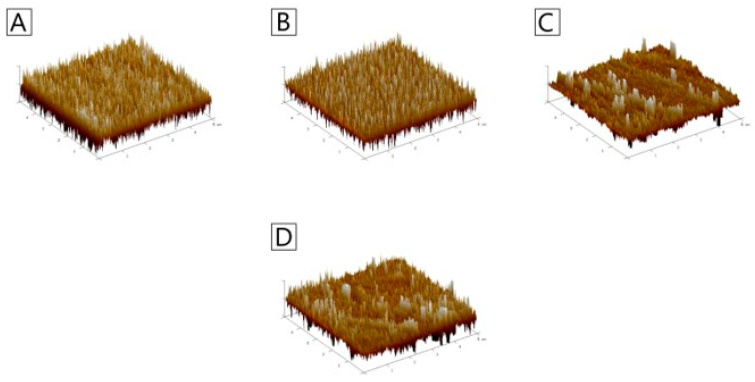
3D images of the surface of (**A**) 100KG, (**B**) 99.75KG/0.25GA, (**C**) 99.50KG/0.50GA, (**D**) 99.00KG/1.00GA (pictures are representative of 3 analyses).

The parameters Ra and Rq represent surface roughness (Table 4; Figure 2), expressed in nanometers. Ra corresponds to the arithmetic average roughness, while Rq refers to the root mean square roughness, which is more sensitive to peaks and valleys on the surface. The reference sample (100KG) exhibited the smoothest surface, with Ra ≈ 0.94 nm and Rq ≈ 1.21 nm. The GA functionalization resulted in a progressive increase in both roughness parameters. A small GA content (0.25%) caused only a minor (statistically non-significant) increase in roughness; however, at 0.50% and 1.00%, both Ra and Rq approximately doubled, showing significant differences compared to the control. The Rq values remained consistently higher than Ra, as expected, since Rq accounts for squared deviations from the mean line. The GA, therefore, leads to a noticeable increase in surface roughness, with significant effects observed at concentrations of 0.50% GA and above. This suggests that higher GA content alters the surface topography, possibly through phase separation, increased crystallinity, or an inhomogeneous distribution of polymer and GA. In summary, the surface roughness parameters (Ra and Rq) increased systematically with GA functionalization, with specimens containing 0.5% and 1% GA showing significant enhancements compared to the control, confirming that GA alters the material’s surface parameters.

### 3.4. DPPH Scavenging Assay

The parameter RSA, representing Radical Scavenging Activity, expresses the antioxidant capacity of the material as a percentage (Table 4). The reference sample (100KG) exhibited minimal antioxidant activity (~0.90%), indicating an almost negligible ability to neutralize free radicals. The GA functionalization resulted in a significant increase in RSA, even at the lowest concentration. Specifically, the 0.25% GA increased RSA by more than twentyfold, from 0.90% to approximately 19.90%. A further moderate increase was observed at 0.50% GA (~21.90%), suggesting that the antioxidant capacity may begin to plateau at this level, while the 1.00% GA formulation showed a remarkable rise to nearly 47%, demonstrating a strong positive correlation. The very low standard deviations (±0.01–0.03) confirm the high reproducibility of the measurements. Overall, the GA significantly enhanced the materials’ antioxidant properties in a dose-dependent manner. In summary, even small additions of GA substantially improved antioxidant performance, while the 1.00% GA specimen exhibited the highest RSA (47.14 ± 0.01%), confirming the strong contribution of GA to the material’s antioxidative functionality.

### 3.5. Water Content

The water content represents the material’s moisture level or its ability to retain water, expressed as grams of water per 100 g of sample (Table 4). The reference sample (100KG) contained approximately 9.65 g/100 g of water. The GA functionalization led to a progressive increase in water content, indicating enhanced hydrophilicity of the material. A 0.25% GA resulted in a modest increase of approximately 15% compared to the control, while at 0.50%, the water content rose significantly to approximately 13.6 g/100 g (around 41% higher than the control). The specimen with 1.00% GA exhibited the highest water content, reaching nearly 16 g/100 g—an increase of about 65%. The differences observed for the 0.50% and 1.00% GA specimens were significant, confirming that higher GA content strongly influences the material’s hydration behavior. This increase in moisture content may enhance the material’s flexibility, softness, and biocompatibility, particularly for biomedical or cosmetic applications such as wound dressings or moisturizing films. In summary, the water content increased systematically with GA functionalization, with the 0.50% and 1.00% GA formulations showing significant enhancement (13.59 ± 1.07 and 15.94 ± 0.48 g/100 g, respectively) compared to the control (9.65 ± 0.41 g/100 g), confirming that GA effectively increases the hydrophilic character and water-retention capacity of the polymeric matrix.

### 3.6. Mechanical Properties

**Figure 3:**
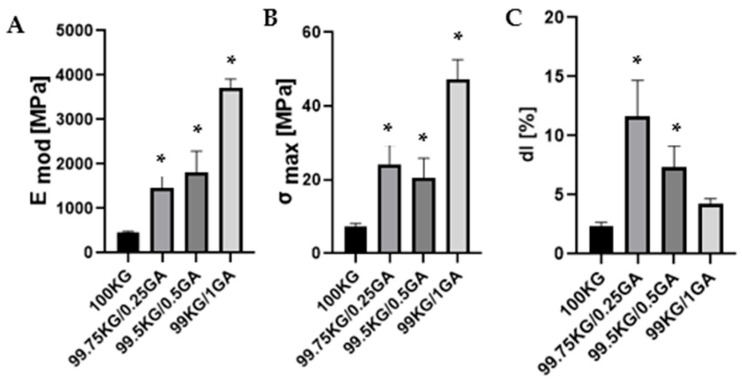
The mechanical parameters of the tested films: Young’s Modulus (**A**), maximum tensile strength (**B**), elongation at break (**C**) (* significantly different from KG-*p* < 0.05).

The GA functionalization had a pronounced effect on the mechanical properties of the materials, as shown in Figure 3. Young’s Modulus increased systematically with GA content, from approximately 500 MPa for the reference sample (100KG) to nearly 4 GPa for the formulation with 1.00% GA, indicating a substantial enhancement in stiffness and rigidity. A similar trend was observed for the maximum tensile strength (σ_max_), which increased from around 10 MPa in the control to over 50 MPa in the 1.00% GA specimens, confirming that GA strengthens the polymeric network. In contrast, the elongation at break (dl) decreased with increasing GA content: while a small GA content (0.25%) improved ductility to about 12–13%, higher concentrations (0.50–1.00%) reduced elongation to 8% and 4%, respectively. These findings demonstrate that GA induces a concentration-dependent increase in mechanical strength and stiffness.

### 3.7. Water Vapor Permeation Rate (WVPR)

The WVPR values of the films are shown in Table 5. The control film (100KG) exhibited the highest WVPR, indicating a low resistance to water vapor transfer. A substantial reduction in WVPR was observed upon GA functionalization, suggesting that it effectively limits water vapor diffusion through the film matrix. The effect was evident even at the lowest GA concentration, where the WVPR decreased markedly compared to the control film. Further increases in GA content enhanced the barrier performance, with the lowest WVPR observed for the 99.00KG/1.00GA film. Overall, these results demonstrate that GA i improves the moisture resistance of KG films in a concentration-dependent manner.

### 3.8. Differences in Color and Whiteness Index

The color and optical appearance of polymeric films are crucial indicators of their composition, homogeneity, and possible interactions between components. In this study, the total color difference (ΔE) and whiteness index (WI) were determined to evaluate the visual changes resulting from the incorporation of gallic acid (GA) into the polymeric matrix. The reference sample (100KG) exhibited the lowest ΔE value (2.90 ± 0.36), indicating minimal deviation from the pure base material and suggesting a high degree of optical uniformity. A slight increase in ΔE was observed for 0.25% GA (3.49 ± 0.74), which may result from partial interactions between GA molecules and the polymeric chains. A more pronounced increase in color difference was noted for the 0.50% GA specimen (ΔE = 4.39 ± 0.43), indicating significant changes in the surface color of the films. Interestingly, the ΔE value for the 1% GA specimen (3.88 ± 0.61) was slightly lower than that for the 0.50% GA, suggesting that higher GA concentrations, color stabilization or self-association of GA molecules may occur, thereby limiting further optical variation. The WI followed a consistent increasing trend with the GA functionalization, from 94.00 ± 0.28 for the control to 95.44 ± 0.64 for the 0.50% GA film.

The color and optical properties of polymeric films provide important insight into material composition, homogeneity, and intermolecular interactions, and are particularly relevant for applications where appearance or optical clarity is important. In the present study, GA functionalization of KG-based films induced measurable but moderate changes in total color difference and whiteness index, indicating that GA modifies the films’ optical characteristics without causing severe discoloration.

### 3.9. In Vitro Biocompatibility

**Figure 4:**
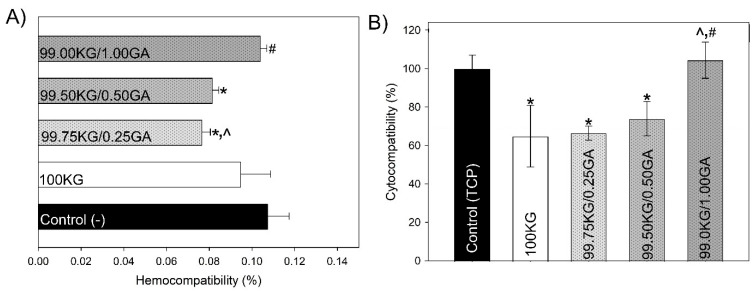
In vitro biocompatibility of tested films: (**A**) hemolytic activity against human erythrocytes after 24 h exposure and (**B**) human fibroblasts viability assessed via MTT assay after 48 h of incubation—indirect test (n = 4; data are expressed as the mean ± SD; * significantly different from the respective controls (*p* < 0.05), ∧ significantly different from the respective film group without GA (*p* < 0.05), # significantly different from the applied concentration of GA (*p* < 0.05).

The results of in vitro biocompatibility assessment are presented in Figure 4. All tested films demonstrated excellent hemocompatibility, with a hemolysis rate below 0.12% after 24 h incubation with human erythrocytes (Figure 4A). This value was markedly lower than the 2% threshold defining non-hemolytic materials [18], as well as below the value recorded for the negative control group, confirming the absence of erythrocyte membrane disruption in all formulations.

In contrast, the cytocompatibility results obtained on human fibroblasts indicated that the GA concentration influenced cell viability (Figure 4B). The unmodified film (KG) and the film modified with a low concentration of GA (0.25%) exhibited a reduction in cell viability to below 70%, thereby classifying them as cytotoxic [22]. Interestingly, increasing the GA from 0.50% to 1.00% markedly enhanced cell survival, suggesting that higher GA concentrations improved cytocompatibility within the KG-based system. Notably, the 99.00KG/1.00GA film exhibited cell viability compared to the TCP control, suggesting that at this concentration, the previously observed cytotoxic effects were effectively mitigated. Collectively, these findings identify this formulation as the most biocompatible and biologically promising candidate for subsequent in vivo evaluation, where its dual antioxidant and biocompatible properties may translate into enhanced tissue-regenerative potential.

## 4. Discussion

This study investigates GA-mediated functionalization as a strategy to modify the properties of KG films. The FTIR analysis revealed only subtle changes in the spectral profiles, indicating that the GA did not fundamentally alter the polysaccharide backbone. Previously, the hydrogen bonds formation was reported in our study of KG functionalized tannic acid [23]. In contrast, studies by Zhang et al. on chitosan modified with phenolic acids reported more pronounced spectral changes indicative of covalent grafting. Comparison with the results reported by Zhang et al. [24] highlights a fundamental difference in the interaction mechanisms governing the two systems. While phenolic-acid-grafted chitosan undergoes chemical modification via covalent amide bond formation, the KG–GA system is stabilized primarily by physical interactions, without disrupting the native polymer structure. This difference may also be related to functionalized conditions—such as pH-mediated changes—as polyphenols can undergo structural transformations, for example, the oxidation of tannic acid (TA) to quinones [25]. Furthermore, such non-covalent interactions may be advantageous for applications that require the preservation of polymer integrity and reversibility. On the other hand, these differences may also arise from the material preparation methods—in the study by Zhang et al., 1-(3-dimethylaminopropyl)-3-ethylcarbodiimide hydrochloride and N-hydroxysuccinimide were applied to graft phenolic acids covalently onto the chitosan backbone.

Surface wettability and surface free energy are key determinants of biomaterial performance, as they directly influence interfacial phenomena such as water interactions, protein adsorption, and cell adhesion [26]. In the present study, the GA functionalization significantly altered their surface wettability and interfacial properties in a concentration-dependent manner. Our studies showed that pristine KG films exhibited moderate contact angles for both glycerol and diiodomethane, reflecting a balanced contribution of polar and dispersive interactions typical of polysaccharide-based materials. These findings are consistent, in part, with previous reports on KG-based systems modified with gluconolactone (GL) or TA [23]. In those systems, the GL increased the total surface free energy and its polar component while reducing the dispersive contribution, which was attributed to its strongly hydrophilic nature. Conversely, increasing TA content decreased both total surface free energy and its polar component due to cross-linking between TA and KG, thereby reducing the number of free hydrophilic groups available at the surface. Compared to these systems, GA-modified KG films exhibit a more nuanced behavior, where surface properties can be finely tuned by controlling GA concentration without inducing extensive cross-linking or excessive hydrophilicity. In contrast to chitosan-based systems modified with carboxylic acids (CA), where CA incorporation increased glycerol contact angles and rendered the films more hydrophobic [18], GA functionalization enhanced surface polarity at low concentrations. Moreover, while dispersive interactions dominated surface energy in CTS/CA systems, the KG/GA films displayed a more balanced interplay between polar and dispersive components, highlighting the distinct role of polymer chemistry and modification strategy in governing interfacial behavior.

Surface roughness is a critical parameter influencing adhesion, wettability, and biological interactions of polymeric films. In the present study, the reference KG film exhibited the smoothest surface, with Ra and Rq values below 1.3 nm, confirming the formation of a uniform and homogeneous polysaccharide matrix. Upon GA functionalization, a systematic increase in both roughness parameters was observed. The consistently higher Rq values relative to Ra reflect a greater presence of surface peaks and valleys, indicating greater topographical heterogeneity. These findings are consistent with previous reports on KG-based systems modified with polyphenolic compounds. For example, films containing TA exhibited progressively higher Ra and Rq values with increasing phenolic content, which was attributed to strong hydrogen-bond interactions between the phenolic groups and KG chains [23]. The behavior of KG/GA films differs from that reported for chitosan systems modified with phenolic acids, where a reduction in surface roughness has often been observed. In chitosan-based materials, phenolic acids can promote chain rearrangement and surface smoothing, particularly when covalent grafting or strong electrostatic interactions occur [27]. The increase in roughness observed in the KG/GA system highlights the importance of polymer chemistry and interaction mechanisms, as KG lacks reactive amine groups and primarily interacts with GA through non-covalent hydrogen bonding.

Antioxidant activity is a key functional property for biomaterials intended for biomedical and protective applications, particularly where oxidative stress plays a critical role. In the present study, pristine KG exhibited negligible radical scavenging activity, reflecting the limited ability of the polysaccharide backbone to donate hydrogen atoms or electrons to neutralize free radicals. In contrast, the GA functionalization resulted in a pronounced and concentration-dependent enhancement of antioxidant capacity, even at very low additive levels. These findings are consistent with those of Wang et al. [23], who reported that grafting phenolic acids to chitosan significantly enhanced antioxidant activity compared to native chitosan. In those systems, the antioxidant performance was strongly dependent on both the type of phenolic acid and the degree of grafting, with gallic acid- and caffeic acid-grafted chitosan exhibiting particularly high radical-scavenging efficiencies. The superior antioxidant activity observed for gallic acid derivatives has been attributed to the high number and favorable arrangement of hydroxyl groups, which promote effective radical neutralization. Despite these similarities, important mechanistic differences exist between the KG/GA system and phenolic-acid-grafted chitosan materials. In chitosan-based systems, the enhancement of antioxidant activity is primarily associated with covalent grafting of phenolic acids onto the polymer backbone, leading to permanent incorporation of phenolic moieties and high radical scavenging efficiencies that can approach those of small-molecule antioxidants such as ascorbic acid [28]. In contrast, the KG–GA films developed in this study rely on non-covalent interactions, such as hydrogen bonding, to immobilize GA within the polymer matrix. Importantly, it should be noted that antioxidant activity based on DPPH assay reflects the chemical radical-scavenging capacity of the material under in vitro conditions and does not directly represent intracellular antioxidant effects [29].

Water content is also a crucial parameter for polymeric films intended for biomedical and cosmetic applications, as it directly influences flexibility, softness, biocompatibility, and wound-healing performance. In the present study, GA functionalization led to a pronounced, concentration-dependent increase in water content, indicating a substantial enhancement of the material’s hydrophilicity and water-retention capacity. These findings are in good agreement with previous studies on KG films modified with TA, where increasing TA content led to higher water content due to the greater availability of hydroxyl groups exposed at the film surface. In those systems, the enhanced affinity for water was directly linked to improved wound-healing potential, as maintaining a moist environment is known to accelerate tissue regeneration [23]. The trend observed here differs from that reported for phenolic-acid–chitosan composite films, in which the incorporation of phenolic acids generally reduced moisture content. In chitosan-based systems, phenolic acids tend to interact strongly with the polymer’s hydrophilic –OH and –NH_2_ groups through hydrogen bonding, effectively reducing the number of free sites available for water binding and leading to lower moisture uptake [30]. In contrast, KG lacks amine functionalities and interacts with GA predominantly through weaker, non-covalent interactions, which appear to preserve or even enhance the accessibility of hydrophilic groups for water sorption.

Mechanical performance is a key determinant of the applicability of polymeric films, particularly for biomedical applications where both strength and flexibility must be carefully balanced. The pure KG film exhibits relatively poor mechanical strength [31]. Its functionalization exerted a pronounced and concentration-dependent improvement in stiffness and tensile strength. A similar trend has been reported for chitosan-based films modified with phenolic acids, where the incorporation of compounds such as ferulic acid led to the highest Young’s modulus values [27]. In both systems, the improvement in stiffness is attributed to enhanced intermolecular interactions between the phenolic acids and the polymer chains, which restrict molecular mobility and reinforce the polymer network. Notably, higher GA concentration resulted in reduced elongation at break, indicating that stronger intermolecular associations increased network rigidity while limiting polymer chain mobility. Such mechanical behavior may still be suitable for many wound dressing applications that require structural stability and barrier protection. Although highly flexible applications, such as dressings intended for dynamic anatomical regions (e.g., joints, elbows, knees, or neck area) may require additional material modification (like plasticizer addition) [32].

Water vapor permeability rate is important to be considered, as it governs moisture transfer across the material. Controlled water vapor permeability is particularly important in wound-healing applications, where maintaining an optimal moist environment can accelerate tissue regeneration, while in packaging, it determines barrier performance and product stability [33,34]. In the present study, WVPR was evaluated using a gravimetric method based on the weight gain of calcium chloride, which directly reflects the amount of water vapor permeating through the film over time. The GA functionalization increases the water vapor permeability compared to the reference KG film. This behavior suggests that GA modifies the polymer matrix to facilitate water vapor transport. The observed increase in WVPR may be attributed to enhanced hydrophilicity of the films resulting from the introduction of hydroxyl and carboxyl functional groups associated with GA, which promote water sorption and diffusion through the matrix. Additionally, GA incorporation may alter the material’s microstructure by increasing free volume or inducing structural heterogeneity, thereby creating more accessible pathways for water vapor migration. These effects are consistent with the observed changes in hydration behavior and permeability. The decrease in WVPR accompanied by an increase in water content suggests that GA induces a denser packing of the polymer matrix, while hydrogen bonding interactions reduce the free volume within the material. The incorporation of GA likely promotes stronger intermolecular interactions between polymer chains, resulting in a more compact, organized structure. As a result, although the material becomes more hydrophilic and capable of absorbing higher amounts of water, the mobility and diffusion pathways for water vapor are restricted. This reduced free volume and increased structural cohesion limit the transport of water molecules through the matrix, thereby enhancing barrier properties despite the higher water uptake. These findings are consistent with reports on chitosan films modified with phenolic acids, where the addition of phenolic compounds increased WVPR relative to pure chitosan [35]. In those systems, higher calcium chloride weight gain and recalculated WVPR values were associated with improved water permeability, indicating that phenolic acid modification can enhance moisture transport properties. In contrast, other studies by Jiang et al. [28] reported a decrease in water vapor permeability upon incorporation of phenolic-rich extracts into chitosan films. This reduction was attributed to strong intermolecular interactions between chitosan functional groups and phenolic compounds, which narrowed diffusion pathways, increased film hydrophobicity, and generated more tortuous routes for water vapor transport. The discrepancy between these observations and the present results highlights the importance of both the nature of the phenolic additive and the interaction mechanism with the polymer matrix.

The color and optical properties of polymeric films provide important insight into material composition, homogeneity, and intermolecular interactions, and are particularly relevant for applications where appearance or optical clarity is critical. In the present study, the GA functionalization induced measurable but moderate changes in total color difference and whiteness index, indicating that GA modifies the optical characteristics of the films without causing severe discoloration. In our previous studies, we reported chitosan/phenolic acid films, where the addition of phenolic acids typically increased ΔE while simultaneously decreasing WI, resulting in darker or more yellow-toned films [35]. The difference in behavior likely reflects fundamental differences in polymer chemistry and interaction mechanisms. In chitosan-based systems, stronger interactions between phenolic acids and amino groups can intensify coloration and reduce brightness, whereas in KG-based films, the absence of amine functionalities may prevent excessive chromophore formation or aggregation.

The developed films, particularly the effects of GA functionalization, were evaluated for biocompatibility. The hemocompatibility results are consistent with the report by Jayeoye et al., in which a pure KG extract was evaluated using a hemolytic assay with rat blood and showed no substantial hemolytic activity at 100 µg/mL [36]. Similarly, the non-hemolytic behavior of GA was previously confirmed [37]. Beyond its blood compatibility, GA also exhibits protective effects on erythrocyte membranes, effectively blocking the access of oxidants and preventing membrane destabilization [38]. Moreover, GA has been demonstrated to inhibit the toxic and hemolytic activity of snake venoms, serving as an active component in functional gels for post-envenomation applications [39]. In contrast, the cytocompatibility indicated that GA concentration influenced cell viability, with higher GA levels improving fibroblast survival. A slight reduction in cell viability was previously reported by Zhu et al. for KG-based hydrogels tested with NIH/3T3 fibroblasts and RAW 264.7 cells; however, the observed decrease remained within the non-cytotoxic range [40]. Furthermore, most available studies have assessed the cytocompatibility of KG within polymeric blends or composite systems, such as KG–chitosan films tested with Chinese hamster ovarian cells [41], KG-silk fibroin sponges [42], or KG–xanthan gum hydrogels with human dermal fibroblasts [43]. These variations may be attributed to differences in the source and physicochemical properties of KG (e.g., molecular weight), the extraction and purification procedures, or even the intended biomedical application. Additionally, the discrepancies may result from the biostability of KG-based materials, as most previous studies have employed KG in blended or chemically modified forms rather than as a standalone matrix [2]. Interestingly, the 1.0% GA functionalization fully mitigated the mild cytotoxicity of KG films, rendering them cytocompatible. This improvement may be attributed to the therapeutic properties of GA (antioxidant—Table 4 and cytoprotective), whose beneficial effects in multiple health conditions have been previously demonstrated [44].

## 5. Conclusions

This study demonstrates that gallic acid (GA) is an effective and versatile modifier of konjac glucomannan (KG) films, enhancing functional properties while preserving the integrity of the polysaccharide backbone. GA functionalization enabled concentration-dependent control over surface wettability, surface free energy, and roughness, highlighting the strong influence of polymer chemistry and interaction type on interfacial behavior. The films exhibited enhanced antioxidant activity even at low GA concentrations, with the highest GA content providing the strongest radical scavenging performance. GA also significantly increased water content and water vapor permeability, indicating improved hydrophilicity and moisture transport—properties that are particularly advantageous for wound-healing and biomedical applications. Mechanical testing revealed marked reinforcement of KG films, with increased stiffness and tensile strength accompanied by reduced flexibility at higher GA concentrations. Optical analysis showed only moderate color changes, maintaining high visual uniformity. Importantly, all formulations were non-hemolytic, while cytocompatibility toward human fibroblasts was strongly dependent on GA content. Based on our studies, KG99.00/GA1.00 films were identified as promising biomaterials, combining antioxidant activity, tunable physicochemical properties, and biocompatibility. This non-covalent modification strategy presented here offers a simple and effective route for developing KG-based materials with potential for further in vivo evaluation and biomedical applications.

## Figures and Tables

**Table 1:** Nomenclature and composition of the different films.

Abbreviation	Sample
100KG	Film based on konjac glucomannan
99.75KG/0.25GA	Film based on konjac glucomannan mixed with gallic acid in a ratio of 99.75/0.25 (*w*/*w*%)
99.50KG/0.50GA	Film based on konjac glucomannan mixed with gallic acid in a ratio of 99.50/0.50 (*w*/*w*%)
99.00KG/1.00GA	Film based on konjac glucomannan mixed with gallic acid in a ratio of 99.00/1.00 (*w*/*w*%)

**Table 2:** The characteristic peaks of all studied films after GA functionalization.

Sample	Wavenumber (cm^−1^)	Sample	Wavenumber (cm^−1^)	Characteristic Group
100KG	~3340	99.75KG/ 0.25GA	~3344	–OH stretching
	~2882		~2884	aliphatic C–H stretching
	~1647		~1639	asymmetric COO^−^ stretching
	~1373		~1374	C–O–C and C–O stretching
	~1245, 1149		~1245, 1148	C–O–C and C–O stretching
	~1018		~1018	C–O stretching
99.50KG/ 0.50GA	~3344	99KG/ 1GA	~3343	–OH stretching
	~2884		~2884	aliphatic C–H stretching
	~1639 (↓)		~1640	asymmetric COO^−^ stretching
	~1374		~1373	C–O–C and C–O stretching
	~1245, 1148		~1244, 1149	C–O–C and C–O stretching
	~1018		~1019	C–O stretching

**Table 3:** The surface free energy (IFT(s)) and polar (IFT (s,P)) and dispersive (IFT (s,D)) components of HA-based and KG-based films with and without phytic acid (n = 5; * significantly different from KG-*p* < 0.05).

Specimen	Θ^G^ [°]	Θ^D^ [°]	IFT (s) [mJ/m^2^]	IFT (s,P) [mJ/m^2^]	IFT (s,D) [mJ/m^2^]
100KG	52.98 ± 1.60	53.02 ± 0.91	41.26 ± 1.48	18.01 ± 0.85	23.25 ± 0.63
99.75KG/0.25GA	52.16 ± 1.86	43.26 ± 0.89 *	44.74 ± 1.25 *	16.35 ± 0.58 *	28.39 ± 0.66 *
99.50KG/0.50GA	50.54 ± 1.54	44.65 ± 0.61 *	45.15 ± 1.14 *	17.85 ± 0.79	27.90 ± 0.41 *
99.00KG/1.00GA	51.40 ± 1.35	50.12 ± 1.40 *	43.50 ± 1.30 *	19.15 ± 0.77	24.35 ± 0.53 *

**Table 4:** The roughness parameters, the antioxidant potential and water content values of KG-based films with and without GA (* significantly different from KG-*p* < 0.05).

Specimen	Ra [nm]	Rq [nm]	RSA [%]	Water Content [g/100 g]
100KG	0.94 ± 0.16	1.21 ± 0.19	0.90 ± 0.01	9.65 ± 0.41
99.75KG/0.25GA	1.04 ± 0.03	1.36 ± 0.06	19.91 ± 0.03 *	11.14 ± 0.28
99.50KG/0.50GA	1.86 ± 0.15 *	2.26 ± 0.19 *	21.87 ± 0.02 *	13.59 ± 1.07 *
99.00KG/1.00GA	1.95 ± 0.13 *	2.46 ± 0.16 *	47.14 ± 0.01 *	15.94 ± 0.48 *

**Table 5:** The water vapor permeability rate (WVPR), total color difference (ΔE) and whiteness index (WI) values for all of the films (n = 3; * significantly different from KG-*p* < 0.05).

Specimen	WVPR	∆E	WI
100KG	0.1159 ± 0.0029	2.90 ± 0.36	94.00 ± 0.28
99.75KG/0.25GA	0.0469 ± 0.0031 *	3.49 ± 0.74	94.68 ± 0.79
99.50KG/0.50GA	0.0426 ± 0.0035 *	4.39 ± 0.43 *	95.44 ± 0.64
99.00KG/1.00GA	0.0354 ± 0.0020 *	3.88 ± 0.61	95.02 ± 0.66

## Data Availability

The raw data supporting the conclusions of this article will be made available by the authors on request.

## Supplementary Materials

The following supporting information can be downloaded at: https://www.mdpi.com/article/10.3390/polym18070832/s1, Figure S1: FTIR-ATR spectra for all studied samples.

## Abbreviations

The following abbreviations are used in this manuscript:

AA	Acetic acid
AFM	Atomic Force Microscopy
ATR-FTIR	Attenuated Total Reflect–Fourier Transform Infrared
DMEM	Dulbecco’s Modified Eagle Medium
DMF	N,N-dimethylformamide
DOAJ	Directory of open access journals
DPPH	2,2-diphenyl-1-picrylhydrazyl
FBS	Fetal bovine serum
GA	Galic acid
KG	Konjac glucomannan
LD	Linear dichroism
MDPI	Multidisciplinary Digital Publishing Institute
MTT	3-(4,5-dimethylthylthiazol-2-yl)-2,5—diphenyltetrazolium bromides
Ra	Arithmetic mean roughness
Rq	Root-mean roughness
RSA	Radical scavenging activity
SDS	Sodium dodecyl sulfate
WVPR	Water Vapor Permeation Rate
TCP	Tissue culture plastic
RBCs	Red blood cells
BJ	Fibroblasts
DMEM	Dulbecco’s Modified Eagle Medium
