# Supplementary figures and images for: Multifunctional Konjac Glucomannan Film Tuning by Gallic Acid Functionalization
Source: Polymers (Basel). 2026 Mar 28;18(7):832. doi: 10.3390/polym18070832 (PMC13074227; doi:10.3390/polym18070832)

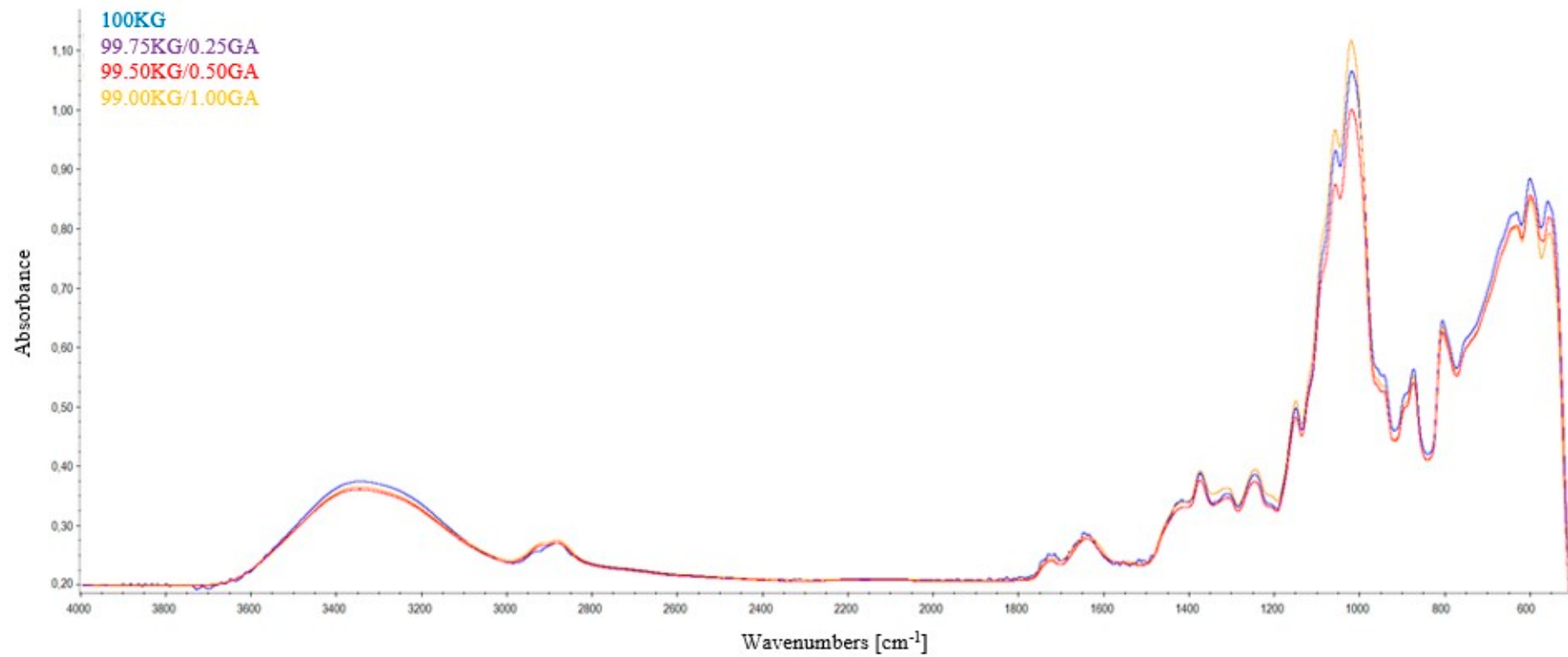

Figure S1. FTIR-ATR spectra for all studied samples

Supplement: Supplementary file 1 [file polymers-18-00832-s001.zip › polymers-4154933-supplementary.pdf]
